# High Risk of Deep Neck Infection in Patients with Type 1 Diabetes Mellitus: A Nationwide Population-Based Cohort Study

**DOI:** 10.3390/jcm7110385

**Published:** 2018-10-25

**Authors:** Geng-He Chang, Meng-Chang Ding, Yao-Hsu Yang, Yung-Hsiang Lin, Chia-Yen Liu, Meng-Hung Lin, Ching-Yuan Wu, Cheng-Ming Hsu, Ming-Shao Tsai

**Affiliations:** 1 Department of Otolaryngology, Chiayi Chang Gung Memorial Hospital, Chiayi 613, Taiwan; genghechang@gmail.com (G.-H.C.); tny4646@gmail.com (M.-C.D.); scm00031@gmail.com (C.-M.H.); 2 Health Information and Epidemiology Laboratory, Chiayi Chang Gung Memorial Hospital, Chiayi 613, Taiwan; r95841012@ntu.edu.tw (Y.-H.Y.); qchiayen@gmail.com (C.-Y.L.); mattlin@cgmh.org.tw (M.-H.L.); 3 Graduate Institute of Clinical Medical Sciences, College of Medicine, Chang Gung University, Taoyuan 33302, Taiwan; 4 Department of Traditional Chinese Medicine, Chiayi Chang Gung Memorial Hospital, Chiayi 613, Taiwan; smbepigwu77@gmail.com; 5 School of Traditional Chinese Medicine, College of Medicine, Chang Gung University, Taoyuan 33302, Taiwan; 6 Division of Endocrinology and Metabolism, Chiayi Chang Gung Memorial Hospital, Chiayi 613, Taiwan; bryam1130@gmail.com

**Keywords:** cervical, cellulitis, abscess, deep neck infection, diabetes mellitus

## Abstract

Objective: To investigate the risk of deep neck infection (DNI) in patients with type 1 diabetes mellitus (T1DM). Methods: The database of the Registry for Catastrophic Illness Patients, affiliated to the Taiwan National Health Insurance Research Database, was used to conduct a retrospective cohort study. In total, 5741 patients with T1DM and 22,964 matched patients without diabetes mellitus (DM) were enrolled between 2000 and 2010. The patients were followed up until death or the end of the study period (31 December 2013). The primary outcome was the occurrence of DNI. Results: Patients with T1DM exhibited a significantly higher cumulative incidence of DNI than did those without DM (*p* < 0.001). The Cox proportional hazards model showed that T1DM was significantly associated with a higher incidence of DNI (adjusted hazard ratio, 10.71; 95% confidence interval, 6.02–19.05; *p* < 0.001). The sensitivity test and subgroup analysis revealed a stable effect of T1DM on DNI risk. The therapeutic methods (surgical or nonsurgical) did not differ significantly between the T1DM and non-DM cohorts. Patients with T1DM required significantly longer hospitalization for DNI than did those without DM (9.0 ± 6.2 vs. 4.1 ± 2.0 days, *p* < 0.001). Furthermore, the patients with T1DM were predisposed to DNI at a younger age than were those without DM. Conclusions: T1DM is an independent risk factor for DNI and is associated with a 10-fold increase in DNI risk. The patients with T1DM require longer hospitalizations for DNI and are younger than those without DM.

## 1. Introduction

Deep neck infection (DNI) is a common infectious disease involving the deep neck space; DNI usually requires intensive care and aggressive treatment [1]. The easy availability of antibiotics, improvements in diagnostic technology, and the concept of early surgical debridement have significantly reduced the morbidity and mortality of DNI [2,3]. However, DNI remains a potentially life-threatening disease when lethal complications, such as descending necrotizing mediastinitis, develop [4,5].

A study reported that patients with diabetes mellitus (DM) are at a 1.4-fold higher risk of DNI than those without DM [6]. DNI can cause higher morbidity and mortality among patients with systemic diseases such as DM, end-stage renal disease, liver cirrhosis, and autoimmune diseases [1,4,7,8,9]. However, the pathogenesis of type 1 DM (T1DM) is different from that of type 2 DM (T2DM). T1DM is characterized by an immune-mediated depletion of beta cells, which causes a lifelong dependence on exogenous insulin [10]. Patients with T1DM, considered to have an immunocompromised status, are expected to be more vulnerable to complicated infection and have a higher infection-related mortality risk than patients with T2DM [11]. Studies investigating the effect of T1DM on DNI are not currently available in the literature. This study investigated the effect of T1DM on DNI occurrence, treatment, and prognosis.

## 2. Methods

### 2.1. Data Source

The government of Taiwan established the National Health Insurance Research Database (NHIRD), which covered 99.6% of Taiwan’s population in 2017 [12,13]. The NHIRD provides all medical claims data of all beneficiaries, including disease diagnoses during clinic visits and hospitalization, prescription drugs and doses, examinations, procedures, surgery, payments, resident locations, and income levels, generated during reimbursement for insurance in an electronic format. The diagnostic codes in the NHIRD are based on the International Classification of Diseases, Ninth Revision, Clinical Modification (ICD-9-CM). This study was exempted from obtaining informed consent from the participants because the data were deidentified. All information of the insurants was unidentifiable, and this study did not violate their rights or adversely affect their welfare. The study was approved by the Institutional Review Board of Chang Gung Memorial Hospital (IRB Number: 201601249B1).

### 2.2. Study Cohort

In Taiwan, T1DM is categorized as a “catastrophic illness” in the NHIRD. Patients with T1DM are certified by the government and included in the Registry for Catastrophic Illness Patients (RFCIP). Therefore, they can avail considerable discounts on medical expenses. The certification process requires critical evaluation of medical records, serological, and pathological reports by experts [14]. Therefore, the T1DM diagnosis of the enrolled patients was highly accurate and reliable.

**Figure 1:**
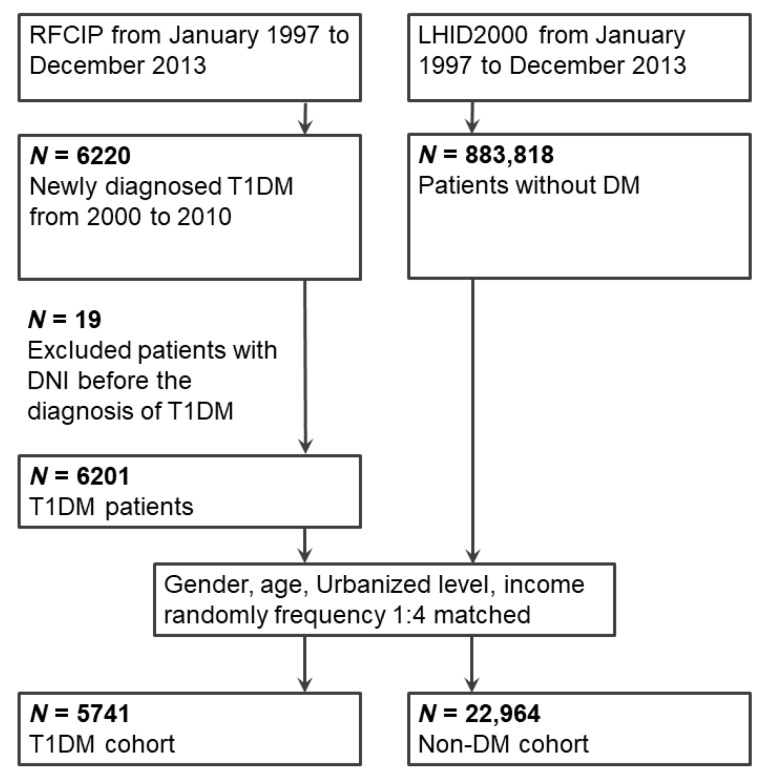
Enrollment schema of the study and comparison cohorts. Abbreviations: RFCIP, Registry for Catastrophic Illness Patients; LHID2000, Longitudinal Health Insurance Database 2000; DM, diabetes mellitus; T1DM, type 1 diabetes mellitus; DNI, deep neck infection.

Data regarding patients who received new diagnoses of T1DM between January 2000 and December 2010 in Taiwan were retrieved from the RFCIP (Figure 1). The patients who received T1DM diagnoses in or after 2011 were not included to ensure a follow-up period of at least 3 years. We used the following T1DM-associated ICD-9-CM codes, which were defined for the RFCIP: 250.01, 250.03, 250.11, 250.13, 250.21, 250.23, 250.31, 250.33, 250.41, 250.43, 250.51, 250.53, 250.61, 250.63, 250.71, 250.73, 250.81, 250.83, 250.91, and 250.93 [14]. In addition, patients who received DNI diagnoses before T1DM were excluded. Finally, 6201 patients with T1DM were enrolled in the study cohort.

### 2.3. Comparison Cohort

The Longitudinal Health Insurance Database 2000 (LHID2000), a subset database of the NHIRD, consists of 1,000,000 insurants who were randomly statistically selected from all insurants in Taiwan in 2000. Age distribution, sex distribution, or health care costs did not differ significantly between the LHID2000 sample group and all enrollees in the NHIRD, according to a report by the National Health Research Institutes [13]. The LHID2000 has been used in several population-based studies [15,16]. We used the LHID2000 to generate a comparison cohort, which consisted of patients without DM.

### 2.4. Matching Process

For each patient with T1DM, four patients without DM were randomly selected from the LHID2000 database, matched for sex, age, urbanization level, and income level to form a comparison cohort. The index date of the study cohort was the date of registry in the RFCIP for patients with T1DM, and an index date matching that of patients with T1DM was created for the comparison cohort. After the matching process, 5741 T1DM and 22,964 non-DM patients were enrolled in the study.

### 2.5. Main Outcome

The main outcome of this study was the occurrence of DNI, which is defined as hospitalization with the following ICD-9 codes: 528.3 (cellulitis and abscess of oral soft tissues; Ludwig angina), 478.22 (parapharyngeal abscess), 478.24 (retropharyngeal abscess), and 682.1 (cellulitis and abscess of neck) [1,17]. The follow-up period was from the index date to the diagnosis of DNI, death, or the end of 2013.

### 2.6. Comorbidities

Comorbidities were defined using ICD-9-CM codes recorded in the claims data: hypertension (HTN) (ICD-9-CM codes: 401–405), cerebrovascular accident (CVA) (ICD-9-CM codes: 430–438), coronary artery disease (CAD) (ICD-9-CM codes: 410–414), chronic kidney disease (CKD) (ICD-9-CM codes: 403, 404, 585, and 586), systemic autoimmune diseases (SADs) (ICD-9-CM codes: 443.1, 446.0, 446.2, 446.4–446.5, 446.7, 696.0–696.1, 710.0–710.4, and 714.0–714.4), and liver cirrhosis (LC) (ICD-9-CM codes: 571.2, 571.5–571.6) [1,17,18,19]. Medical comorbidities were included if they appeared at least once in the diagnoses of inpatients or at least thrice in the diagnoses of outpatients.

### 2.7. Treatment Modalities

The treatment methods were divided into two subgroups: “surgical” and “nonsurgical.” The patients who received surgical intervention were included in the “surgical” subgroup, whereas those who received antibiotic or abscess aspiration without surgery were included in the “nonsurgical” subgroup [1].

### 2.8. Prognosis Evaluation

For evaluating prognosis, we analyzed the duration of hospitalization, care in intensive care units (ICUs), performance of tracheostomy, and mediastinal complications, which were defined according to the receipt of mediastinal surgery during hospitalization or the diagnostic codes of mediastinitis (ICD-9-CM codes: 510, 513, and 519.2) [1]. Mortality and mediastinitis-related mortality were also investigated in both cohorts. Mortality was defined as death occurring during DNI treatment. Mediastinitis-related mortality was defined as death during DNI treatment accompanied by the diagnosis of mediastinitis [1]. In addition, we analyzed the age distribution of the patients with DNI identified in the T1DM and non-DM cohorts.

### 2.9. Statistical Analysis

The demographic characteristic and comorbidities of the T1DM and non-DM cohorts were compared using the Pearson’s chi-square test for categorical variables and the unpaired Student *t*-test for continuous variables. Control variables, such as age, sex, urbanization level, income level, and comorbidities (HTN, CVA, CAD, CKD, SADs, and LC) were included as covariates in the univariate model. Variables in the univariate analysis that showed *p* < 0.1 were included in the multivariate analysis. Kaplan–Meier analysis was used to estimate the cumulative incidence in the two cohorts, and the differences were determined using a two-tailed log-rank test. Multivariable Cox proportional hazard regression models were used to measure the hazard ratio (HR) and 95% confidence interval (CI) of DNI incidence between the T1DM and non-DM cohorts. In addition, the stability of HR was examined using sensitivity testing and subgroup analysis if the interaction effects between the comorbidities and T1DM on DNI were significant. All analyses were performed using SAS software, version 9.4 (SAS Institute, Cary, NC, USA), and the level of statistical significance was set at *p* < 0.05.

## 3. Results

Table 1 illustrates the distribution of sociodemographic characteristics, DNIs, and comorbidities identified in the T1DM and non-DM cohorts. The T1DM cohort exhibited a significantly higher prevalence of DNI, HTN, CVA, CAD, CKD, SADs, and LC. Among the 5741 patients with T1DM, 42 (0.7%) patients with DNI were identified, and the incidence rate was 92.4 per 100,000 person-years in a mean follow-up period of 7.91 ± 2.41 years. By contrast, among the 22,964 controls, 16 (0.1%) patients with DNI were identified in a mean observation period of 8.08 ± 2.29 years, and the incidence rate was 8.6 per 100,000 person-years. The incidence rate ratio was 10.73 with a 95% CI of 6.03–19.08. The incidence of DNI was significantly higher in the T1DM cohort than in the non-DM cohort (*p* < 0.001).

**Figure 2:**
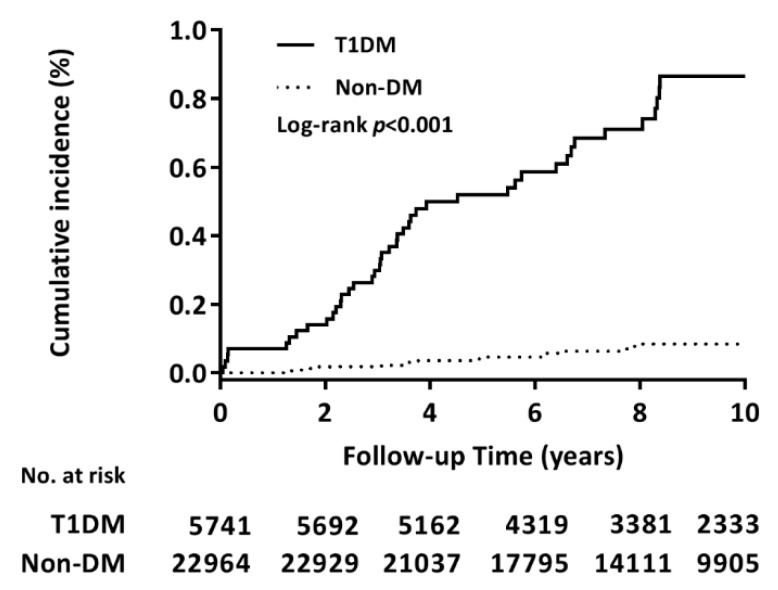
Cumulative incidence of DNI in the T1DM versus non-DM cohorts. Kaplan–Meier analysis demonstrated the cumulative DNI identified in the T1DM and non-DM cohorts during the 10-year follow-up period. The log-rank test revealed a significantly higher cumulative incidence in the T1DM cohort than in the non-DM cohort (*p* < 0.001).

Results of the Kaplan–Meier analysis revealed the cumulative incidence of DNI in both the cohorts over a 10-year observation period. The T1DM cohort exhibited a significantly higher incidence of DNI than the non-DM cohort did (log-rank test *p* < 0.001, Figure 2). The Cox proportional hazards model revealed that T1DM was associated with a 10-fold higher risk of DNI (adjusted HR: 10.71, 95% CI: 6.02–19.05, *p* < 0.001, Table 2). In addition, the sensitivity test showed a stable effect of T1DM on DNI risk in the study cohort in the main model with each additional covariate. The results of subgroup analysis showed that T1DM is a risk factor for DNI in all the subgroups.

Table 3 presents the treatment modalities and prognosis of DNI in the patients in both cohorts (Table 3). Although the percentage of patients requiring surgical treatment for DNI was higher in the T1DM cohort than in the non-DM cohort, the difference in the percentages was not significant (T1DM vs. non-DM cohorts = 33.3% vs. 18.8%, *p* = 0.276). DNI in the patients in the T1DM cohort required longer hospitalization durations than did those in the non-DM cohort (T1DM vs. non-DM cohorts: 9.0 ± 6.2 vs. 4.1 ± 2.0 days, *p* < 0.001). Furthermore, care in ICU and mediastinal complications were only identified in patients with T1DM and DNI (ICU: 6/42, 14.3%; mediastinitis: 1/42, 2.4%). DNI-related mortality was observed in the T1DM cohort (mortality: 2/42, 4.8%) but not in the non-DM cohort.

**Figure 3:**
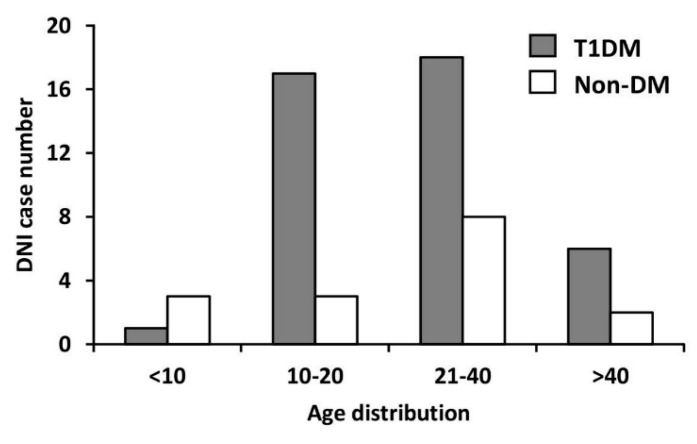
Age distribution of DNI in the T1DM and non-DM cohorts. The peak age of DNI occurrence in the non-DM cohort was 21–40 years, while those in the T1DM cohort were 10–20 years and 21–40 years.

Figure 3 presents the age distribution of DNI identified in the T1DM and non-DM cohorts. We divided the age into the following four groups: <10, 10–20, 21–40, and >40 years. Accordingly, the proportions of DNI in the two cohorts (T1DM vs. non-DM) were 2.38% vs. 18.75% (<10 years), 40.47% vs. 18.75% (10–20 years), 42.86% vs. 50% (21–40 years), and 14.28% vs. 12.5% (>40 years). In this study, the peak age of DNI occurrence in the non-DM cohort was 21–40 years, while the T1DM cohort exhibited two peak ages, namely 10–20 and 21–40 years.

## 4. Discussion

Our nationwide study is the first to examine the influence of T1DM on DNI. Our study demonstrated that T1DM is a definite risk factor for DNI. Our results revealed that patients with T1DM are at a 10-fold higher risk of DNI than were those without DM. The higher frequency of infections in patients with T1DM is attributable to hyperglycemia, which results in immune dysfunction, including disrupted neutrophil function, depression of the antioxidant system and humoral immunity, micro- and macroangiopathies, neuropathy, decrease in the antibacterial activity of urine, gastrointestinal and urinary dysmotility, and the need for medical intervention in these patients [20].

Patients with T1DM are more likely to have complicated infections, such as pneumonia, septicemia, and osteomyelitis, than are those without DM [21]. Simonsen et al. reported that the incidence of bacterial infections was significantly higher in patients with T1DM than in those without DM [22]. Muller et al. reported that patients with T1DM and T2DM have an increased risk of infections of the lower respiratory tract, urinary tract, and skin and mucous membranes [23]. In addition, an Australian diabetes register-based study revealed that patients with T1DM exhibited significantly higher infection-related mortality (pneumonia, septicemia, and osteomyelitis) than did those with T2DM [11]. Therefore, T1DM is a risk factor for complicated infections, and it might be associated with higher incidence and severity of infection than T2DM.

Previous studies have reported that surgical treatment was used in 55–80% of patients with DNI [4,9,24,25,26,27,28]. In our study, few patients received surgical treatment in both the cohorts (T1DM: 33.3% and non-DM: 18.7%). This difference in percentage may result from previous studies being conducted in medical centers or tertiary hospitals, which receive and treat patients with severe DNI [4,8,9,24,25,26,27]. Hence, patients with severe DNI were more likely accept surgical interventions. However, we enrolled patients from all hospitals in our nationwide study. The distribution of patients with DNI was from primary to tertiary hospitals, and patients with low DNI severity were also included; thus, our study provided a complete spectrum of DNI treatment and prognosis [1]. In general, the use of surgical interventions to treat a DNI indicates that the infection is more severe and life-threatening. In our study, the percentage of surgical treatment for DNI was higher in the T1DM cohort than in the non-DM cohort; however, the difference was not statistically significant.

DNIs in patients with DM have been reported to be associated with long hospitalization durations and numerous complications [4,28,29,30]. In our study, the duration of hospitalization for DNI was significantly higher in the T1DM than in the non-DM cohort, and this result was consistent with previously reported findings. Patients with T1DM and DNI were reported to exhibit a higher frequency of lethal complications, such as mediastinitis (2.7–10.0%), and higher mortality (1.6–7.5%) than those without DM [4,28,29]. A higher rate of ICU care for DNI was noted in patients with T1DM than with those without DM [1]. In our study, the occurrence of ICU care for DNI, mediastinitis, and DNI-related mortality was higher in the T1DM cohort than in the non-DM cohort, and these results were consistent with those of previous studies.

We analyzed the age distribution of DNI in the T1DM and non-DM cohorts in our study. In the non-DM cohort, the peak age of DNI occurrence was 21–40 years, while in the T1DM cohort, the two peak ages of DNI occurrence were 10–20 years and 21–40 years. In addition, in the T1DM cohort, DNI developed at age 10–20 years. In general, the incidence of DNI was higher at age 20–40 years. Patients with diabetes have been reported to have a late onset of DNI [24,25,28,31]. However, T1DM was characterized by diagnosis at a young age; according to Magliano’s report, infection (pneumonia, septicemia, and osteomyelitis) at a young age was more likely to occur in patients with T1DM than in patients with T2DM (T1DM vs. T2DM = 26.9 vs. 60.4 years) [11]. In summary, we believe that patients with T1DM tend to develop DNI at younger age (10–40 years) than do patients without DM (21–40 years) and those with T2DM (>40 years).

Our study has several strengths, including a large number of patients with T1DM representing a nationwide population and a 10-year observation period. In addition, the diagnosis of T1DM was based on data from the RFCIP, a highly accurate and reliable database affiliated to the NHIRD. Nevertheless, the study has some limitations. The diagnoses were based on ICD-9-CM codes and not on original medical records; therefore, it lacked blood sugar level, laboratory data, data from imaging studies, surgical records, and pathologic reports, which are necessary for evaluating disease severity. The bacterial spectrum and drug sensitivity of T1DM-DNI and the difference from non-DM-DNI are important information for clinical management and prescription of antibiotics. However, our database did not contain that information. The effects of the factors omitted in this study on T1DM and DNI should be investigated in future studies. In addition, ICU care, mediastinitis, and mortality were observed only in patients with T1DM; however, the number of patients was insufficient to develop a statistical conclusion. Additional studies including detailed medical records and a large sample size of patients with DNI are needed.

## 5. Conclusions

This nationwide population-based study was the first to investigate the epidemiological data of DNI development and prognosis in patients with T1DM. We concluded that T1DM is a predisposing factor for DNI. The duration of hospitalization for DNI is longer in patients with T1DM than in those without DM. In addition, patients with T1DM are predisposed to developing DNI at a younger age than are those without DM.

## Figures and Tables

**Table 1:** Demographic characteristics of the T1DM and non-DM cohorts.

Characteristic	T1DM		Non-DM		*p*-Value *
	*N*	%	*N*	%	
**Total**	5741		22,964		
**Gender**					1
Male	2777	48.4	11,108	48.4	
Female	2964	51.6	11,856	51.6	
**Age (years)**					1
<20	2751	47.9	11,004	47.9	
≥20	2990	52.1	11,960	52.1	
**Urbanized level**					1
1 (City)	1616	28.2	6464	28.2	
2	2662	46.4	10,648	46.4	
3	867	15.1	3468	15.1	
4 (Village)	596	10.4	2384	10.4	
**Income (NTD, per month)**					1
0	4005	69.8	16,020	69.8	
1–15,840	608	10.6	2432	10.6	
15,841–25,000	731	12.7	2924	12.7	
≥25,001	397	6.9	1588	6.9	
**Comorbidities**					
HTN	1073	18.7	1192	5.2	<0.001
CVA	227	4.0	350	1.5	<0.001
CAD	340	5.9	435	1.9	<0.001
CKD	332	5.8	111	0.5	<0.001
SADs	139	2.4	388	1.7	<0.001
LC	118	2.1	89	0.4	<0.001
**DNI**					
Total	42	0.7	16	0.1	<0.001

Abbreviations: T1DM, type 1 diabetes mellitus; NTD, New Taiwan dollar; HTN, hypertension; CVA, cerebrovascular accident; CAD, coronary artery disease; CKD, chronic kidney disease; SADs, systemic autoimmune diseases; LC, liver cirrhosis; DNI, deep neck infection. * Pearson’s chi-square test.

**Table 2:** Multivariable Cox proportional hazards model for associations between DNI and T1DM.

Variables	HR	95% CI	*p*-Value
Main model *	10.71	(6.02–19.05)	<0.001
**Additional covariates †**			
Main model + HTN	10.20	(5.66–18.38)	<0.001
Main model + CVA	10.54	(5.91–18.77)	<0.001
Main model + CAD	10.67	(5.99–19.04)	<0.001
Main model + CKD	10.36	(5.79–18.56)	<0.001
Main model + SADs	10.66	(5.99–18.97)	<0.001
Main model + LC	10.27	(5.75–18.33)	<0.001
**Subgroup effects**			
**Gender**			
Male	4.69	(2.12–10.38)	<0.001
Female	24.67	(8.51–71.52)	<0.001
**Age**			
<20	14.28	(5.74–35.56)	<0.001
≥20	6.88	(3.07–15.39)	<0.001
**Without selected comorbidity**			
HTN	10.03	(5.40–18.63)	<0.001
CVA	9.41	(5.17–17.14)	<0.001
CAD	9.34	(5.13–17.00)	<0.001
CKD	9.47	(5.22–17.20)	<0.001
SADs	9.27	(5.09–16.88)	<0.001
LC	9.67	(5.32–17.56)	<0.001

* Main model was adjusted for sex, age, urbanized level, and income. † The model was adjusted for sex, age, urbanized level, income, and each additional comorbidity. Abbreviations: DNI, deep neck infection; T1DM, type 1 diabetes mellitus; NTD, New Taiwan dollar; HTN, hypertension; CVA, cerebrovascular accident; CAD, coronary artery disease; CKD, chronic kidney disease; SADs, systemic autoimmune diseases; LC, liver cirrhosis.

**Table 3:** Treatment modalities, complications, and prognostic outcomes in patients with DNI.

Characteristic	T1DM-DNI		Non-DM-DNI		*p*-Value
	*N*	%	*N*	%	
**Total**	42		16		
**Therapy**					0.276 ^a^
Non-surgery	28	66.7	13	81.3	
Surgery	14	33.3	3	18.8	
**Tracheostomy**	0	0.0	0	0.0	
**Hospitalization (day, mean ± SD)**	9.0 ± 6.2		4.1 ± 2.0		<0.001 ^b^
**ICU care**	6	14.3	0	0.0	
**Mediastinitis**	1	2.4	0	0.0	
Mediastinitis-Mortality	1	2.4	0	0.0	
Mortality *	2	4.8	0	0.0	

* Mortality occurrence after DNI. ^a^ Pearson’s chi-square tests. ^b^ Student *t*-tests. Abbreviations: DNI, deep neck infection; SD, standard deviation; ICU, intensive care unit.

## Data and Material Availability

The datasets generated and/or analyzed during the current study are available in the Taiwan National Health Insurance Research Database repository [32].
